# Family and home correlates of television viewing in 12–13 year old adolescents: The Nepean Study

**DOI:** 10.1186/1479-5868-3-24

**Published:** 2006-09-10

**Authors:** Louise L Hardy, Louise A Baur, Sarah P Garnett, David Crawford, Karen J Campbell, Vanessa A Shrewsbury, Christopher T Cowell, Jo Salmon

**Affiliations:** 1 NSW Centre for Overweight and Obesity, University of Sydney, Australia; 2 The Children's Hospital at Westmead, Australia; 3 Discipline of Paediatrics & Child Health, University of Sydney, Australia; 4 Centre of Physical Activity & Nutrition Research, Deakin University, Australia

## Abstract

**Background:**

Few young people meet television viewing guidelines.

**Purpose:**

To determine the association between factors in the family and home environment and watching television, including videos and DVDs, in early adolescence.

**Methods:**

Cross-sectional, self-report survey of 343 adolescents aged 12–13 years (173 girls), and their parents (338 mothers, 293 fathers). Main measures were factors in the family and home environment potentially associated with adolescents spending ≥ 2 hours per day in front of the television. Factors examined included family structure, opportunities to watch television/video/DVDs, perceptions of rules and regulations on television viewing, and television viewing practices.

**Results:**

Two-thirds of adolescents watched ≥ 2 hours television per day. Factors in the family and home environment associated with adolescents watching television ≥ 2 hours per day include adolescents who have siblings (Adjusted Odds Ratio [95%CI] AOR = 3.0 [1.2, 7.8]); access to pay television (AOR = 2.0 [1.1, 3.7]); ate snacks while watching television (AOR = 3.1 [1.8, 5.4]); co-viewed television with parents (AOR = 2.3 [1.3, 4.2]); and had mothers who watched ≥ 2 hours television per day (AOR = 2.4 [1.3, 4.6]).

**Conclusion:**

There are factors in the family and home environment that influence the volume of television viewed by 12–13 year olds. Television plays a central role in the family environment, potentially providing a means of recreation among families of young adolescents for little cost. Interventions which target family television viewing practices and those of parents, in particular, are more likely to be effective than interventions which directly target adolescent viewing times.

## Background

Television is ubiquitous, with at least one television set present in most family homes in developed countries [1]. It is, therefore, not surprising that time spent in front of a television set watching television, videos or DVDs is one of the most prevalent leisure time activities among young people in industrialized nations [2-4]. While there are potential benefits from watching some television programs, videos and DVDs there are concerns that time spent in front of a television, particularly during daylight hours, displaces more cognitively and physically challenging pursuits [5]. The evidence that physical activity among children and adolescents is displaced by time spent in front of television is, however, equivocal [6]. Nevertheless, there is consistent evidence that time spent in front of a television is associated with a number of negative outcomes among adolescents, including overweight and obesity [7] (although it has been argued that this relationship is not clinically significant, [8], poor dietary habits [9], and social problems (e.g., aggressive behavior, low school achievement) [3].

Guidelines for television viewing recommend that children aged 2 years and older watch less than two hours of television per day [10]. However, large proportions of children and adolescents in many countries do not meet these recommendations. In developed countries, between 25% and 40% of adolescents aged 11 to 15 years report watching more than 3–4 hours per day [4].

Intervention efforts to reduce adolescents' television/video/DVD viewing need to be informed by an understanding of the influences on television viewing among young people. Social ecological models of health behavior [11] provide a framework for examining how socio-cultural forces, social institutions, and family and environment influence television viewing behavior. The family environment, in particular, plays a potentially important role in children's development of health behaviors because parents are children's key sources of influence [12]. Factors in the family environment that have the potential to influence the time children spend in front of a television include parenting practices [13,14], family characteristics, [15,16], family television viewing practices and access to television [17,18].

Limited research exists on the influence of the family environment and time spent in front of televisions during early adolescence [17,18]. In this study we sought to identify factors in the family environment that influence television, video, and DVD viewing in early adolescence. The factors investigated included the adolescents' and parents' self report on family structure, opportunities to watch television/video/DVDs, perceptions of rules and regulations on television viewing, and television viewing practices, including eating habits in front of the television.

## Methods

### Participants

Between July 2002 and February 2003, 434 adolescents aged 12–13 years, who participated in the longitudinal Nepean Study (1996 to 1998), were followed up in the Nepean Kids Growing Up Study. The adolescents were part of a birth cohort born between August 1989 and April 1990, at Nepean Hospital, in western Sydney (Australia), and their details have been previously published [19,20]. An additional five students who were part of the original birth cohort but who did not participate in the Nepean Study volunteered to participate in this phase of data collection after publicity about the study. Of the 343 (79%) participants, 331 attended an interview and 12 completed the entire study at home and returned their questionnaires by mail. Of the 91 subjects not followed up, 46 were not contactable and 45 declined to participate. Three-hundred and thirty-eight mothers and 293 fathers of the adolescents participated in the study.

### Design and ethics

This cross-sectional study focused on adolescents' television video and DVD viewing, and how this activity is influenced by factors in the family and home environment. The Ethics Committees of The Children's Hospital at Westmead and the Wentworth Area Health Service gave ethical approval for this study. Written consent was obtained from the adolescents' mother or father and each adolescent signed a study agreement form.

### Measures

Questionnaires were mailed to participating families to complete (one for adolescents and one for the parents collectively). Participants were instructed to complete the surveys at home and to bring the completed questionnaires to a follow-up interview at the study centre. Parents and adolescents were interviewed separately and each questionnaire checked with the respondent for completeness and clarification of any doubtful responses.

### Socio-demographic information

The adolescents' parents reported the socio-demographic characteristics of the family, including marital status, employment status, parental education and number of children in household. For the analyses, marital status was classified as single or dual parent family. The age and sex of each child under 21 years of age in the household was recorded. Socio-economic status (SES) was measured using maternal education and categorized into low (did not complete high school), middle (completed high school or trade certificate), and high (tertiary education). Maternal education was used as an indicator of SES because maternal education is linked with child health, where mothers with higher education being more likely to engage in health promoting behaviors [21].

### Adolescent Questionnaire

Adolescents completed a questionnaire that included behavioral and environmental items relating to television viewing. The reliability of the adolescent questionnaire was assessed using a 2-week test-retest method on a separate sample of adolescents (n = 140). Individual items showed good to excellent repeatability (percent agreement = 70% – 99%).

### Television, video and DVD usage

Adolescents were asked to report how much time they spend watching television/videos/DVDs on a usual school day and on a usual weekend day. Average daily time spent in front of the television screen was calculated and, for the analyses, were classified according to the recommended guidelines for television viewing that is, either less than 2 hours/day or ≥ 2 hours/day [10].

### Television technology in the home

Adolescents were asked to complete an inventory of items in their home that may encourage television usage including the number of televisions in their home, television in their bedroom, video player, DVD player and Pay Television.

### Practices associated with television/video/DVD viewing

Adolescents were asked to think about the past few months and report how often they engaged in the following practices whilst sitting in front of a television: ate dinner, ate snacks, co-viewed with their mother or father; ate dinner with their mother or father, and ate snacks with their mother or father. The response categories were: never or rarely, less than once/week, about 1–3 times/week, about 4–6 times/week, and everyday. For analyses, the categories were collapsed into <once/week and ≥ once/week to indicate seldom and habitual practices.

### Adolescents' perceptions of parents' beliefs and rules on television use

Adolescents were asked to respond to the following statements, each starting with, *My mother and/or father *"...try to make sure that I do not watch too much television"; "...switch off the television if they think I am watching too much"; "...let me watch television when I do something good"; and "...think that if they did not monitor my activity levels I would watch too much television". Response categories were: disagree; slightly disagree; neutral; slightly agree; and agree. For analyses, the categories were collapsed into disagree/neutral and agree. Neutral responses were categorized with disagree responses, because they indicate there were no definitive perception of rules regarding television viewing.

### Parents' questionnaire

#### Parents' television, video and DVD usage

Using a previously validated instrument [22] mothers and fathers were each asked to estimate the total time (hours or minutes) that they spent watching television (including videos and DVDs) during the previous week. Average daily time spent in front of the television screen was calculated separately for mothers and fathers, and for the analyses were dichotomized into <2 hours/day or ≥ 2 hours/day.

#### Parents' rules and restrictions for television viewing

One parent was asked to complete a multiple choice questionnaire assessing parenting styles. Items asked parents to indicate whether they allowed their child to watch any television show they choose; restricted how much time their child spends watching television; prohibited their child from watching television until their homework is done; and did not permit the television to be on during meal times. These questions were derived from a previously reliability tested and published instrument [23]. Parents were also asked whether their partner shared the same views regarding how much television their child watches; and whether their partner supported rules about when their child can watch television. Response categories were: disagree; slightly disagree; neutral; slightly agree; and agree. For analyses, the categories were collapsed into disagree/neutral and agree.

### Data analysis

Data were analyzed using Statistical Package for Social Science (SPSS) version 12.01 [24]. Descriptive statistics were used to characterize participating adolescents and their families. For the analysis, time spent watching television/videos/DVDs was categorized according to the recommended guidelines for watching television [10], that is <2 hrs or ≥ 2 hrs per day. All explanatory variables were dichotomized to indicate whether practices were seldom or habitual and to provide definitive responses for perception of rules. Bivariate analyses were conducted using the chi-squared (χ^2^) continuity correction statistic and tests for linear trend (p < 0.05). Unadjusted odds ratios (OR) were calculated to quantify the risk associated with individual explanatory variables.

The independent effects of the predictor variables were calculated by entering significant bivariate associations, commencing with the largest estimate, stepwise into multiple logistic regression models to estimate the adjusted OR and 95% confidence intervals (CI). As sex, SES, marital status and parents' employment status were not associated with adolescents' television viewing, they were not included in the multivariable model. Multi-collinearity between explanatory variables was assed using Spearman's rank correlation co-efficient and change (i.e., >10%) in the standard errors in the logistic regression model.

## Results

### Descriptive

The characteristics of the families included in these analyses (343 adolescents; 338 mothers; 293 fathers) are given in Table 1. The parent questionnaires were completed mainly by mothers (90%). The sample comprised adolescents from middle-class families (65%) with two parents (88%) and siblings (97%). Twenty-four percent of mothers and 81% of fathers were in full time paid employment. Half (49.6%) of the adolescents were male and the mean age was 12.9 years.

**Table 1:** Descriptive characteristics of adolescents and family (n = 343)

**Variable**	**Prevalence**	**N**
Age (year; mean ± SD)	12.9 ± 0.21	343
Boys	49.6%	170
**Family characteristics**		
Dual parent	88%	301
Mother in full time paid employment	24%	57
Father in full time paid employment	81%	243
Siblings	93%	320
Socioeconomic status		
Low	10%	34
Middle	65%	224
High	25%	85
**Time spent watching television/video/DVDs**		
*Boys*		
Hours per day (mean ± SD)	2.5 ± 1.3	170
≥ 2 hours/day (%)	66%	112
*Girls*		
Hours per day (mean ± SD)	2.6 ± 1.3	173
≥ 2 hours/day (%)	66%	114
*Mother*		
Hours per day (mean ± SD)	1.4 ± 1.3	340
≥ 2 hrs/day	27%	93
*Father*		
Hours per day (mean ± SD)	1.8 ± 1.4	295
≥ 2 hrs/day	41%	122
**Home television environment**		
Number of TV sets in the home (mean ± SD)	2.9 ± 1.6	343
Video recorder	98%	337
DVD player	52%	179
Television in the bedroom	36%	124
Pay television	26%	90

Two-thirds of the adolescents reported spending more than 2 hours a day watching television/video/DVDs, with boys watching a median of 2.3 hours per day (range 0 – 5.7 hrs/day) and girls 2.4 hours per day (0 – 7.6). Less than one-third of mothers and 40% of fathers watched ≥ 2 hours television/video/DVDs per day.

The prevalence of explanatory variables and unadjusted OR for adolescents who spent more than 2 hours/day watching television and factors associated with families and the home environment that may promote television viewing are shown in Table 2. The unadjusted OR in Table 2 show that the strongest predictors for adolescents watching ≥ 2 hours television per day were having a mother who watched ≥ 2 hours television per day (p < 0.001), and co-viewing television with parents (p < 0.001). Subscriptions to pay television (p < 0.02), eating snacks or dinner in front of the television without parents (p < 0.001), and eating snacks or dinner in front of the television with parents (p < 0.02 and p < 0.03, respectively) were also significantly related. Parents' rules and adolescents' perceptions of parents and beliefs and rules for television/video/DVD viewing were not significant predictors of adolescent television viewing time.

**Table 2:** Family and home environment and likelihood of adolescents watching television ≥ 2 hours/day.

**Explanatory variables**	**Prevalence (%) ≥ 2 hrs TV/day (n)**	**OR (95% CI)†**
**Family characteristics**		
Socioeconomic status		
Low	76.5 (26)	1.0
Middle	65.2 (146)	0.6 (0.3, 1.3)
High	62.7 (52)	0.5 (0.2, 1.3)
**Parental status**		
Dual parents	66.1 (199)	1.0
Single parent	64.3 (27)	0.9 (0.5, 1.8)
**Siblings**		
No	47.8 (11)	1.0
Yes	67.2 (215)	2.2 (1.0, 5.2)
**Family television/video/DVD viewing practices**		
*Mother*		
< 2 hrs/day	60.2 (148)	1.0
≥ 2 hrs/day	79.3 (73)	2.5 (1.5, 4.5)*
*Father*		
< 2 hrs/day	62.4 (108)	1.0
≥ 2 hrs/day	70.0 (84)	1.4 (0.9, 2.3)
*Adolescent watches television with parents*		
< 1 time/week	47.3 (35)	1.0
=> 1 time/week	71.2 (190)	2.8 (1.6, 4.7)**
**Televisions in the home**		
*Televisions in home*		
< 3	62.3 (149)	1.0
≥ 3	73.8 (76)	1.7 (1.0, 2.8)
*Television in bedroom*		
No	63.0 (138)	1.0
Yes	70.7 (87)	1.4 (0.9, 2.3)
*Pay television*		
No	62.1 (157)	1.0
Yes	76.4 (68)	2.0 (1.1, 3.4)*
*DVD*		
No	60.4 (99)	1.0
Yes	70.8 (126)	1.6 (1.0, 2.5)
**Adolescent eating practices associated with television/video/DVD viewing**		
*Eats dinner in front of television*		
< 1 time/week	58.1 (104)	1.0
≥ 1 time/week	74.7 (121)	2.1 (1.3, 3.4)*
*Eats snacks in front of television*		
< 1 time/week	44.1 (41)	1.0
≥ 1 time/week	73.8 (183)	3.6 (2.2, 5.9)*
*Eats dinner with parents in front of television*		
< 1 time/week	60.7 (119)	1.0
≥ 1 time/week	72.6 (106)	1.7 (1.1, 2.7)*
*Eat snacks with parents in front of television*		
< 1 time/week	61.1 (135)	1.0
≥ 1 time/week	74.2 (89)	1.8 (1.1, 3.0)*
**Adolescents' perception of parents' beliefs & rules**		
*My parents make sure I don't watch too much television*		
Disagree	63.9 (69)	1.0
Agree	65.3 (111)	1.1 (0.6, 1.8)
*My parents switch off the television if they think I am watching too much*		
Disagree	65.4 (104)	1.0
Agree	73.9 (88)	1.5 (0.9, 2.5)
*My parents let me watch television when I do something good*		
Disagree	66.3 (110)	1.0
Agree	65.6 (63)	1.0 (0.6, 1.7)
*My parents monitor my activity levels so I wouldn't watch too much television*		
Disagree	66.3 (126)	1.0
Agree	64.9 (61)	0.9 (0.6, 1.6)
**Parents' rules for adolescent's television/video/DVD viewing‡**		
*I do not allow the television on during meal times*		
Agree	70.2 (66)	1.0
Disagree	72.4 (118)	1.0 (0.9, 1.2)
*I allow my child to watch any television show he/she chooses*		
Disagree	63.1 (154)	1.0
Agree	69.4 (43)	1.3 (0.7, 2.4)
*I restrict how much time my child spends watching television*		
Disagree	73.2 (30)	1.0
Agree	63.0 (133)	0.6 (0.3, 1.3)
*My child is not allowed to watch television or play electronic games until his/her homework is done*		
Disagree	68.2 (30)	1.0
Agree	64.5 (136)	0.9 (0.4, 1.7)
*My partner and I have the same views about how much television our child should watch*		
Disagree	60.0 (12)	1.0
Agree	63.6 (145)	1.2 (0.5, 3.0)
*My partner supports the rules I make about when our child is able to watch television*		
Disagree	56.3 (9)	1.0
Agree	64.1 (152)	1.4 (0.5, 3.9)

† Odds ratios (95% confidence intervals) derived from chi-squared analysis; ‡ Parent report

*P < 0.05; **P < 0.001

The best predictive model (Table 3) of the family and home environment and adolescents spending ≥ 2 hours per day in front of a television explained 20% of the variation and included adolescents with siblings (p = 0.02); who have access to pay television (p = 0.03); co-viewing television with parents (p = 0.004); a mother who watches ≥ 2 hours television per day (p = 0.01); and eating snacks in front of the television (p < 0.001).

**Table 3:** Logistic regression correlates for family and home environment and adolescents watching television/video/DVDs ≥ 2 hours/day.

**Explanatory variables**	OR (95% CI)*	**P value**
Has siblings	3.0 (1.2, 7.8)	0.02
Mother watches television/videos/DVDs ≥ 2 hrs/day	2.2 (1.2, 4.1)	0.01
Adolescent watches television with parents ≥ 1/week	2.3 (1.3, 4.1)	0.004
Access to Pay television	2.0 (1.1, 3.7)	0.03
Eats snacks in front of television set ≥ 1/week	3.5 (2.1, 5.9)	0.001

* Odds ratios (95% confidence intervals) are adjusted for all variables in the model.

## Discussion

The aim of this study was to examine the association between the family and home environment and adolescent television/video/DVD viewing. Two-thirds of adolescents in this study spent ≥ 2 hours a day in front of the television, which is above recommended guidelines [10]. Our findings indicate that adolescents with siblings, a mother who views more than 2 hours television/video/DVDs a day, co-view with their parents, have access to Pay Television, and eat snacks in front of the television are more likely to watch ≥ 2 hours television per day. Among this sample of adolescents, aspects of the family and home environments appear to be related to the amount of television watched.

The odds of adolescents spending ≥ 2 hours a day in front of a television doubled when they co-viewed with parents, and trebled in families with more than one child. Other studies have also reported associations between adolescents' and parents' rates of television viewing [25-27], suggesting that parents have a significant influence on their offspring's television viewing habits. The context in which families view television/video/DVDs is complex, but it is feasible that time spent co-viewing with parents is considered a shared social activity and may serve as a means of bonding between young adolescents and parents. Dubas and colleagues reported that parents tend to increase co-viewing as children move into early adolescence, while less time is spent together in other social contexts (e.g., eating together, going out) [28].

Alternatively, the association may be because parents are concerned about the content of television and co-viewing allows parents to monitor television viewing. However, the rules assessing parental monitoring of adolescent television viewing in the current study did not distinguish between those participants exceeding or not exceeding recommended viewing time, perhaps because of the age of the participants. Families represent an important environmental influence in the development and maintenance of healthful habits among children; and efforts to reduce television viewing among adolescents will most likely need to have a family focus that targets reduced viewing among all family members.

Twenty-seven per cent of mothers and 41% of fathers reported watching ≥ 2 hours of television per day; however, only mothers' television viewing was significantly associated with adolescents' viewing more television than recommended. Parents are considered the primary agents and gatekeepers of children's development, socialization and well-being. Research among infants shows that a substantial number of children begin watching television during pre-school years and that these early viewing patterns persist into child and adolescent years [14]. Qualitative research suggests that television viewing plays an important role in assisting busy mothers cope with young children, and many value television as a good educational tool, a 'babysitter/coping mechanism', and a medium to either stimulate or calm down preschoolers [29]. Potentially, early childhood exposure to television viewing establishes later television viewing behavior, which in turn may be incorporated into family lifestyles. A recent tracking study confirms this theory, with children in the top quartile for television viewing at 6 years of age, four times more likely to be in the top quartile at 9 years [30].

Several studies [17,18,31], but not all [23,32] have reported that a having a television set in children's bedrooms is associated with higher levels of television viewing. Television sets were present in 36% of the adolescents' bedrooms in this study, but having a television in the bedroom was not significantly associated with viewing time. This is an important finding because it suggests that factors in the family environment, such as siblings and family viewing practices may be better indicators of young adolescents at risk of exceeding national television/video/DVD viewing guidelines than television opportunities in the home. Few studies have examined the association between pay television and increased television viewing [23,32,33]. Subscribing to pay television is a parental decision which determines not only what is available to the family to view, but an alternative, low cost means of family entertainment. In this study, access to pay television was indicative of high television viewing among adolescents.

In the current study, adolescents who ate snacks while watching television were more than three times more likely to watch ≥ 2 hours per day. The prevalence of eating snacks while watching television increases with age [34], and is associated with an increases in energy intake and decreased fruit and vegetable consumption [35,36]. Research on the influence of food advertising shows that advertising during the time that early adolescents typically watch television is predominately for micronutrient-poor energy dense foods, and the time spent watching television is linked to adolescent's consumption of these foods, including higher intakes of energy, fat, sweet and salty snacks, and carbonated beverages [37]. Potentially, the co-existence of snacking behavior may also be likely to be cued by either television viewing, and/or the snack (particularly after school) and be a prompt to switch on the television. If this is the case, then interventions targeting snacking behavior, rather than television viewing, may be more successful in reducing both caloric intake and time spent viewing television.

Family meal time is an important component of the family environment because it offers opportunities for the family to interact and has been associated with a healthier dietary intake in adolescents [38,39]. In our study watching television during meal-time, either alone or with parents, was significant at a bivariate level, but not when included in the multivariate model. Other studies have shown television viewing during family meal time to be positively related to television viewing hours [17,18,23,40]. Developing strategies to switch off the television during meal times may be one approach for reducing overall viewing time, and may also reduce associations between television and food (both snacking and meals), which is possibly a significant contributor to overweight and obesity.

There were several limitations to this study including the cross-sectional design which prohibits determining the causal relationship between factors in the family and home environment and adolescents' television viewing. Both the parent and adolescent questionnaires have acceptable face validity, but neither were validated against criterion (i.e., observation) variables. While the use of self-report instruments can be problematic, particularly among children and adolescents, the test-retest reliability of the adolescent questionnaire used in this study demonstrated good to very good repeatability. The majority of adolescents in this study lived in two parent families which is usual in Australia [41], however because the sample comprised mainly middle and high SES families the generalisability of the results is limited.

There is mounting evidence that excessive television viewing among children and adolescents can seriously challenge young people's emotional and physical well-being. Our results add to the limited research that has examined factors in the family and home environment that are associated with excessive television viewing during early adolescence. The current study shows there are strong associations between the family unit and the time young adolescents spent in front of the television. Because parents are a key influence on children's behavior, interventions that target family television viewing practices, parents in particular, are more likely to be effective than interventions which directly target adolescent viewing times.
